# Insight and Dissociation in Lucid Dreaming and Psychosis

**DOI:** 10.3389/fpsyg.2018.02164

**Published:** 2018-11-12

**Authors:** Ursula Voss, Armando D’Agostino, Luca Kolibius, Ansgar Klimke, Silvio Scarone, J. Allan Hobson

**Affiliations:** ^1^Psychology, Johann Wolfgang Goethe-Universität Frankfurt am Main, Frankfurt, Germany; ^2^VITOS Hochtaunus Klinik, Psychiatrisches Krankenhaus, Friedrichsdorf, Germany; ^3^Department of Health Sciences, Università degli Studi di Milano, Milan, Italy; ^4^Department of Psychiatry, Psychiatry Heinrich-Heine-Universität Düsseldorf, Düsseldorf, Germany; ^5^Beth Israel Deaconess Medical Center, Harvard Medical School, Boston, MA, United States

**Keywords:** REM sleep, dreaming, consciousness, EEG, psychosis, delirium, lucid dreaming, affective disorder

## Abstract

Dreams and psychosis share several important features regarding symptoms and underlying neurobiology, which is helpful in constructing a testable model of, for example, schizophrenia and delirium. The purpose of the present communication is to discuss two major concepts in dreaming and psychosis that have received much attention in the recent literature: insight and dissociation. Both phenomena are considered functions of higher order consciousness because they involve metacognition in the form of reflective thought and attempted control of negative emotional impact. Insight in dreams is a core criterion for lucid dreams. Lucid dreams are usually accompanied by attempts to control the dream plot and dissociative elements akin to depersonalization and derealization. These concepts are also relevant in psychotic illness. Whereas insightfulness can be considered innocuous in lucid dreaming and even advantageous in psychosis, the concept of dissociation is still unresolved. The present review compares correlates and functions of insight and dissociation in lucid dreaming and psychosis. This is helpful in understanding the two concepts with regard to psychological function as well as neurophysiology.

## Introduction

REM sleep dreaming and psychosis share several important features such as hallucinogenic imagery, reduced metacognitive thought and disturbed reality discrimination. Moreover, both dreams and psychosis proceed under a lower level of consciousness, characterized by a sense of presence – dreams are immersive, they involve *here and now* experience – and an absence of future-oriented planning and reflection of past experience (Hobson and Voss, 2010; Joli, 2011). Usually, both the dreamer and also the psychotic patient assume a first-person perspective, experiencing themselves as active agents instead of passive by-standers. However, dreams and also psychotic episodes may be accompanied by varying degrees of insight and subjective control. They may also include dissociative phenomena such as depersonalization and derealization or out-of-body experiences^1^.

Insight, control, and dissociation represent the defining criteria of lucid dreams (Van Eeden, 1913; Voss et al., 2009; Voss and Hobson, 2014). Insight is considered the core criterion, whereas control and dissociation are often, but not always enhanced in lucid dreams. Control enables dreamers to alter the dream plot, dissociation occurs when the dreamer experiences the dream as if it was displayed on a screen, as in waking derealization, or sees himself from the outside, as in waking depersonalization (Sierra and Berrios, 2001; Falkai, 2015; see also Windt and Voss, 2018).

Similar to insight gained in lucid dreams in healthy adults, some psychotic patients also acquire insight into the internal nature of their hallucinations or into the illness itself. Such insight has been shown beneficial for therapeutic outcome and is associated with higher quality of life and a better prognosis (Watson et al., 2006; Lincoln et al., 2007; Boyer et al., 2012; Chan et al., 2012; Riggs et al., 2012). Likewise, perceived control over psychotic illness is associated with less co-morbid depression (Birchwood et al., 1993) and need for care (Peters et al., 2017). Dissociation is also common in psychosis (Allen et al., 1997; Moskowitz et al., 2005; Touskova et al., 2018). Its functionality, though, especially with regard to coping with illness, is less clear. Mostly, the concept of dissociation serves to differentiate psychotic from neurotic thought (Bürgy, 2010; Peters et al., 2017).

The purpose of this review is to discuss the prospects of self-induced manipulation of conscious states and to explore the boundaries of possible therapeutic interventions in psychosis. We will give special consideration to the role of lucid insight and dissociation and debate the transferability of research findings from lucid dreams to insightful psychosis.

## Consciousness in Dreams and Psychosis

Dream consciousness arises during REM sleep, in the context of a weakened sensorimotor attachment to the external environment (Windt, 2018). The complex and dynamic experience of dreaming is typically characterized by the loss of insight into its internal origin (Nir and Tononi, 2010). Due to an attenuated activation of the prefrontal cortex during REM sleep, dreamers are deprived of their ability to think logically or to make meaningful decisions (Hobson, 1999; Maquet, 2000, Maquet et al., 2005; Voss et al., 2009; Dresler et al., 2012; Torterolo et al., 2016). However, they are intensively engaged in the dream experience, which is often overwhelmingly emotional. The dream feels real although it isn’t, and this misperception bears high similarity with psychotic and delirious states (Freud, 1900/2011; Hobson, 1999; Scarone et al., 2007; Feinberg, 2011; Joli, 2011).

In previous publications (Hobson and Voss, 2010, 2011; Voss et al., 2011), we have referred to dream consciousness as primary, originally based on Freud’s (1900/2011) understanding of primary processes to describe “a *mode of cognition* that is characterized by a primitive, animistic style of thinking” (Carhart-Harris and Friston, 2010, p. 1266. See also Sinclair, 1922; Edelman, 2004). Primary consciousness represents a lower level of consciousness and is meant to describe a sense of immediate presence and an absence of future-oriented planning and reflection of the past: “Primary consciousness is all that is present to the subject from moment to moment in one unitary block, or it is the continuous succession of such presences, before reflection, judgment or reasoning have set in; before there is any consciousness of consciousness.”… [Secondary consciousness] … is the awareness of awareness. It includes all reflection, judgment, inference, inductive, and deductive reasoning, all intellectual processes of experiment and discovery, even such immediacy as the flash of scientific intuition. It is the play of thought round and about its object.” (Sinclair, 1922, pp. 112–113). In other words, secondary consciousness requires meta-cognitive thought. We will also refer to it as higher order consciousness.

Regarding dream consciousness, we consider it to be mostly primary (Hobson and Voss, 2010, 2011). Carhart-Harris et al. (2014) described it as “regressive style of cognition that is qualitatively different to the normal waking consciousness of healthy adult humans” (p. 6). In this primary mode of consciousness, dreamers are deprived of the means to control and influence the ongoing experience. Their only choice is to cope with the immediate and constantly changing scenery. Consciousness in psychosis has been described in similar terms (e.g., Galderisi et al., 2004; Joli, 2011; Limosani et al., 2011), involving alterations of the sense of agency, unity and continuity that evolve subtly from the prodromal stage to full-blown psychotic disorders (Schneider, 1957; Galderisi et al., 2004; Nelson et al., 2009; Hauser et al., 2011). Psychotic patients suffer from altered perception and processing of reality and impaired self-reflectiveness (Bora et al., 2007), marking psychotic states as mostly primary. Often, patients are not able to distinguish between internal and external cues^2^.

We consider lucid dreaming and insightful psychosis as *hybrid* because in both states thinking is only partially ruled by lower level consciousness. To some extent, dreamers or patients have – however, limited – access to higher order consciousness, enabling them to reflect on their present state. The dream is still a dream, and the hallucination remains a phantasm. However, lucid dreamers are able to distance themselves from the ongoing imagery and may even be successful in attenuating its emotional impact.

Neurochemically, both dreams and psychotic/delusional states have been found to occur against a hypercholinergic and hypo-aminergic background (Hobson and McCarley, 1977; Ciprian-Ollivier and Cetkovich-Bakmas, 1997; Hobson, 1999, 2009; Molina et al., 2005; Minzenberg et al., 2009; Brooks et al., 2010; Gottesmann, 2011), with dopaminergic changes mediating the wake/sleep state dependency (Perry et al., 1999; Collerton and Perry, 2011; Llewellyn, 2011). These neurochemical modulations mainly affect the basal forebrain and mesopontine regions (Woolf and Butcher, 2011). Electrophysiologically, REM sleep dreams and psychotic states are accompanied by strongly attenuated activation and synchronicity in the gamma frequency band (Voss et al., 2009; Gandal et al., 2012; McNally and McCarley, 2016; Reilly et al., 2018), suggestive of reduced conscious awareness and executive ego functions (Desmedt and Tomberg, 1994; Cavinato et al., 2015). By contrast, a relative increase in gamma band activity is observed in lucid dreaming (Voss et al., 2009, 2014), and it would be interesting to explore if similar patterns can be identified in insightful psychoses.

## Lucid Dreams and Dissociative Mental States

Lucidity in dreams refers to a peculiar mental state in which the dreamer is aware of the fact that he is dreaming while the dream continues. We have referred to this conscious awareness as “insight” (Voss et al., 2013). To be able to reflect on the state of arousal, namely to know that one is asleep and dreaming, requires that one can take on a third person perspective, i.e., to look at oneself as if through the eyes of an outsider. We label this kind of thinking “dissociative.” Dissociative thought is heightened in lucid dreaming as compared to normal REM sleep dreaming. In lucid dreams, taking on a third person perspective often entails not only the dreamer as a person but the dream experience itself. Dreamers then report to have seen the dream sequence from the outside, almost as if the dream were an ongoing theatrical production or motion picture (Noreika et al., 2010; Voss et al., 2012, 2014). We consider this to reflect an even higher level of dissociative thought. In other words, lucid dreams can be considered dissociated states of consciousness in which the dream self separates from the ongoing flow of mental imagery. This phenomenological dissociation is physiologically accompanied by highly selective increases in gamma band activity in fronto-temporal areas of the brain (Voss et al., 2009, 2014; Dresler et al., 2012). At the same time, occipito-parietal regions retain a typical REM-sleep profile.

Thus, whereas normal REM-sleep dreams share common neurobiological substrates with organic and so-called “functional” psychoses (Hobson, 2004; Scarone et al., 2008), lucid dreams represent a condition of the brain/mind akin to dissociative mental states in waking, such as derealization and depersonalization. These terms are used to define subjective experiences of detachment from the environment or from one’s experience of oneself or body, respectively (American Psychiatric Association [APA], 2000; Simeon and Abugel, 2006). In waking, dissociative thought is generally transient and can be experienced as an isolated phenomenon or within the context of several different psychiatric diagnoses (Moskowitz et al., 2005). In healthy adults, dissociative thought is present, for example, in daydreaming. It has been linked to fantasy proneness and vivid experiences during the day (Watson, 2001; Giesbrecht et al., 2007). In psychotic awake patients, dissociative thought is a common symptom strongly associated with anxiety (Allen et al., 1996), loosening the moorings in inner and outer reality, thus hampering orientation in the here-and-now. In patients suffering from posttraumatic stress disorder (PTSD), dissociation and especially depersonalization is thought to protect from emotional pain through excessive downregulation of limbic system activity (Lanius et al., 2010) via enhanced activation in the ventral prefrontal cortex (VPFC) (Phillips et al., 2001; Felmingham et al., 2008) as well as altered midcingulate and insula activation (Phillips et al., 2001; Ludaescher et al., 2010). Thus, dissociative thought in these patients can be viewed as a self-protective process, enabling self-conscious emotions through VPFC activation (Damasio, 1988) and suppressing unconscious affect arising from limbic system arousal (see Forgas, 2008).

Electrophysiologically, dissociative thought has been shown to be associated with a suppression in the α band and raised levels of θ activity (Giesbrecht and Merckelbach, 2006) and with a defective communication between the frontal and temporal lobes (Ford et al., 2005; Bob et al., 2010). Despite efforts to explain these experiences in terms of underlying neurobiology (Frewen and Lanius, 2006), it is still common in clinical practice to conceptualize them in psychodynamic terms. In psychoanalytic terminology, these would be symptoms of neurotic (Freud, 1937), borderline (Kernberg, 1967) or immature (Vaillant, 1994) defense mechanisms that fend off undesired wishes and desires of the Id in waking. Cognitively oriented clinical psychology tends to interpret them as a consequence of abuse or traumatic experiences which cause a loss of inhibitory control over affective memories, leading to the emergence of “split” mental contents in response to negative emotional stimuli (Yates and Nasby, 1993).

Dissociative states have often been discussed in the context of positive symptoms in psychotic illness, which mark theses states as undesirable. However, recent studies suggest that dissociative thought is perhaps better understood as transient in the early disease stages which later may – or may not – lead to a loss of sense of self and depersonalization (Dalle Luche, 2002). In this sense, the dissociative phenomena observed in lucid dreaming mirror those in the early disease stages of psychosis. Regarding normal REM-sleep dreaming vs. lucid dreaming, the observed increase in dissociative thought might then represent a weakening of psychosis-like phenomena which marks dissociation in dreams as desirable. In clinical terms, dissociative thought in lucid dreams may serve to exit the quasi-psychotic state of dreaming, whereas dissociative thought in patients at risk may provide the inlet for full-blown psychosis. In both states, lucid dreaming and psychosis, dissociative thought appears to be an interim phenomenon which we refer to as *hybrid*. An interesting question is whether lucid dreaming, and with it dissociative thought, can be used to alleviate psychotic symptoms in wake patients.

Regarding the function of dissociative thought, it seems to be accompanied by a down-regulation of negative emotion in dreaming as well as in psychiatric disorders (LaBerge and Rheingold, 1991; Phillips et al., 2001; Voss et al., 2013; Gackenbach and Bosveld, 2014). Several studies show lucidity in dreams to be associated with positive rather than with negative emotions (LaBerge and Rheingold, 1991; Voss et al., 2013; Gackenbach and Bosveld, 2014), albeit most studies do not differentiate between positive feelings *during* the dream vs. positive feelings *following awakening* from a lucid dream. It is our observation (Voss et al., unpublished) that dreamers often experience their lucid dreams to be emotionally neutral, sometimes accompanied by a sense of achievement (“I did it!” and “success!”) and euphoria after awakening, both instances falling under the category of what Lewis (1995) refers to as “self-conscious emotion.” Self-conscious emotions such as guilt, shame, or empathy require the ability to reflect on the self and, therewith, frontal lobe as well as anterior insular cortex (AIC) activity (Gu et al., 2013).

By contrast, emotionality in normal REM sleep dreams resembles what is known as “unconscious affect” (Oehman and Soares, 1994; Clore and Ketelaar, 1997; Berridge, 1999; Kihlstrom et al., 2000; Winkielman and Berridge, 2004), referring to “valenced good/bad reactions that occur in the absence of conscious awareness” (Winkielman and Berridge, 2004, p. 193). Neurobiologically, unconscious affect is primarily a subcortical process (Morris et al., 1998).

In normal REM sleep dreams, the VPFC is down-regulated, allowing for unconscious affect to prevail (Hobson, 1992). By contrast, lucid dreaming – through reactivation of the VPFC, seems to increase self-conscious emotions and a down-regulation of unconscious affect (Clore and Ketelaar, 1997), resulting in reduced negative (and perhaps overall) emotionality compared to normal dreams. Thus, emotional experience accompanying dissociative thought, namely a strengthening of self-conscious affect and a repression of unconscious emotion appear similar in psychiatric illness and in lucid dreaming.

However, dissociation might represent a stumbling block in the direct transference of lucid dreaming techniques on treatment options for psychosis. In an individual treatment attempt, Voss and Klimke (unpublished) recently tried to apply gamma frequency band electrical stimulation of the fronto-temporal parts of the brain in an acute psychotic patient with acoustic hallucinations^3^. This method has previously been successfully used to induce lucidity in dreams of healthy young adults (Voss et al., 2014), resulting in enhanced ratings of insight and dissociative thought. The respective patient reported to normally hear a voice constantly degrading and insulting him, also often telling him to harm himself and others by jumping out of windows or to physically attack caretakers. He perceived the voice as real and was not able to distance himself from it. Following his 4th stimulation session with 40 Hz, he still heard the voice but now he was able to “negotiate” with this voice so as to prevent self-harm. In one instance, when his voice told him to do a back flip from his bedside table, the patient refused, telling his voice that this would be “not sensible.” Instead, he jumped down from the table feet first. Following his 5th session, however, he experienced extreme frightfulness, asking to be restrained and to be put under surveillance. The treatment was immediately terminated and the patient was able to recover.

While this particular case study must not be over-generalized, it shows that (1) insight can indeed be triggered via mechanisms akin to lucid dreaming and (2) that increased insight and dissociative thought may not automatically effect positive emotionality. Whereas in non-psychotic patients, insight and dissociative are linked with reduced negative emotions, the opposite may apply to psychotic patients (see also Allen et al., 1996).

## Correlates of Lucid Dreaming

**FIGURE 1:**
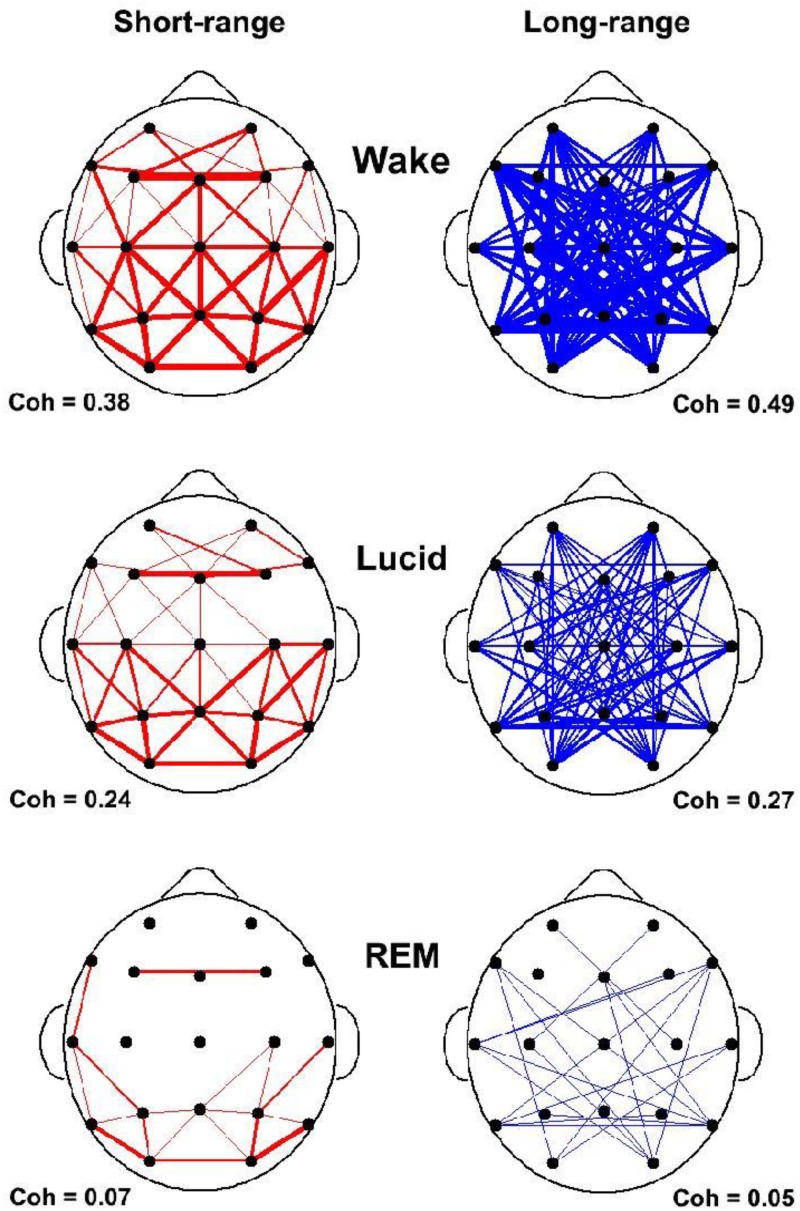
State-dependent coherences in the 40 Hz frequency band. Short and long range coherences obtained for waking (top), lucid dreaming (middle) and REM sleep (bottom). Coherences are indications of interscalp networking and synchronization. Coherences are averaged across electrode pairs in 4 s epochs. Short-range (55 pairs) was defined as less than 10 cm and long-range (65 pairs) as larger than 15 cm inter-electrode distance. Coherences are lowest in REM sleep and strongly enhanced in lucid dreaming.

**FIGURE 2:**
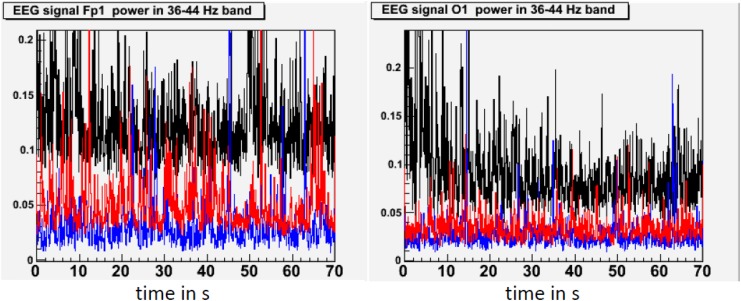
State-dependent power in the 40 Hz frequency band. This figure shows standardized 40 Hz power (36–44 Hz) in waking (black), lucid dreaming (red) and REM sleep (blue) in a single subject. 40 Hz band activity was chosen because effects of lucid dreaming were greatest here. Activity in the 40 Hz band has often been linked with conscious information processing in waking. Topographic images are based on movement-free EEG episodes and are corrected for ocular artifacts. For each state, power values are averaged across the respective episode. Depicted averages are based on episodes of equal duration. Compared to REM sleep, lucid dreaming shows increased 40 Hz activity across the entire cortex. The increase is strongest in frontal regions (left frame) and weakest in occipital regions (right frame).

In our study of EEG tracings during lucid dreaming, the most striking finding was that lucidity was accompanied by an increased activation of the frontal lobes of the brain (see Figures 1, 2). This applies both to synchronicity (Figure 1) and to consciousness-related frequencies (40 Hz, Figure 2). In a study on the formal features of lucid dreaming, we have learned that the most unique characteristic of lucidity is insight into the delusional character of the dream (Voss et al., 2013). We take this to be a clue to a new approach to treatment, one that reinforces frontal lobe function explicitly. We know that the spontaneous occurrence of lucid dreaming is especially frequent during childhood and puberty (Voss et al., 2012), a time in which we experience the final stages of frontal lobe myelination, and a time of synapse expansion and dendritic growth. These neurobiological changes provide the prerequisites for the integration of the frontal lobes into the cortico-cortical and cortico-thalamic networks (Goldman-Rakic, 1987; Zilles et al., 1988; Fuster, 1989). Lucid dreaming may thus occur naturally during the final stages of frontal lobe integration, a process similar to an update of computer hardware. It appears that the peak in spontaneous dream lucidity in childhood and puberty is nothing more than an accidental confounding of conscious states during a time of high cerebral diversification.

An intriguing finding is that lucid dreaming and, through it, dissociation, can be trained. Interestingly, dissociative phenomena like derealization and depersonalization can also easily be trained in the laboratory during this same period in ontogenetic development (Leonard et al., 1999).

## Clinical Implications of Lucidity

As can be seen in Table 1a, lucid dreaming is physiologically distinct from dreaming and schizophrenic psychosis in that lucidity is accompanied by frontal lobe activation and increased synchronicity between the frontal and occipito-parietal parts of the brain. As to neurobiology, at present, we can only speculate that it may be less cholinergically and more aminergically modulated than non-lucid dreaming. With regard to cognitive functions, imagery in lucid dreaming is as bizarre as it is in dreaming and psychosis (Table 1b).

**Table 1:** Physiology and cognitive functions in lucid dreaming, REM sleep dreaming, and schizophrenia.

	Lucid dreaming	Dreaming	Schizophrenia
**(a) physiology**			
Frontal lobe activation	**+**	-	-
Synchronicity	**+**	-	-
Cholinergic innervation	**?**	**+**	**+**
Aminergic innervation	**?**	-	-
**(b) dream-like cognitive functions**			
Delusion	**+**	**+**	**+**
Bizarreness	**+**	**+**	**+**
Awareness	**+**	**+**	**+**
Amnesia	**+**	**+**	-
Disorientation	**+**	**+**	-
**(c) wake-like cognitive functions**			
Awareness of awareness	**+**	-	-
Planning behavior	**+**	-	-

Example 1: *“I realize I am in a dream and think of things to do. Since it is dark and cloudy in the dream, I decide to let the sun shine. It works, but then the sun is shining and it is still raining.” (Voss, unpublished).*

Intentional properties such as directing awareness and performing plot control are significantly enhanced in lucid dreaming as compared to either non-lucid dreaming or psychosis (Table 1c).

In dreams and psychosis alike, internally generated stimuli that rise to consciousness are interpreted as external signals generated by the environment. Weakening of abstract thinking and loss of metacognitive thought (i.e., reflective thinking) appear to determine impaired reality testing in both conditions, suggesting a crucial role for the frontal lobes. In lucidity, reactivation of these cerebral regions during REM sleep can explain insight into the hallucinatory nature of one’s experience. Intriguingly, frontal lobe processing is known to play a critical role within the complex neural circuitry underlying lack of insight in psychotic disorders (Antonius et al., 2011). Disruption of synaptic plasticity in the prefrontal cortex has been confirmed in rodent models of schizophrenia, and available antipsychotic medications are known to modulate LTP in this area (Goto et al., 2010). Cognitive Remediation Therapy, which contributes to the reduction of psychotic symptoms, has also been shown to enhance activity in the prefrontal cortex of schizophrenic patients (Wexler and Bell, 2005).

Regarding maturational influences, clinically relevant symptoms of the so-called major psychoses are known to emerge during adolescence or early adult life. Neurodevelopmental hypotheses converge on abnormal pruning or apoptosis of prefrontal synapses and fibers as a possible pathogenetic mechanism for the complex alterations in higher functions found in these disorders (Bossong and Niesink, 2010).

Given this type of experimental evidence, it has been speculated that dream lucidity might prove beneficial in fostering insight in psychotic patients (see Dresler et al., 2015). Indeed, one’s awareness of the internally generated hallucinatory and often bizarre nature of the dream could be considered the physiological equivalent of a psychotic patient’s increase in insight. Already, dream lucidity has been proposed by some authors as a possible therapeutic intervention in patients affected by nightmare-related disorders such as PTSD (Brylowski, 1990; Zadra and Pihl, 1997; Spoormaker and van den Bout, 2006; Been and Garg, 2010). However, a recent study by Mota et al. (2016) did not confirm the hypothesis that lucid dreams and psychotic episodes are mirror images of each other. Psychotic patients reported lucid dreaming episodes as often as non-psychotic controls, and psychotic lucid dreamers had as severe psychiatric symptoms as non-lucid psychotic dreamers. Regarding control, lucid dreamers claimed to have even more control over the dream plot than non-psychotic controls. Although the authors did not explicitly assess dissociative elements in dreams and waking in psychotic patients, our own clinical observation (Voss and Klimke, unpublished) suggests that it is the dissociative thought and not insight in dreams which these patients experience as unsettling. It reminds them of their deficits in waking thought so that they associate rather negative affect to lucid dreams. So far, experimental evidence in favor or against this assumption does not exist. We look forward to future studies investigating the different aspects of lucidity in dreams and their neurophysiologic correlates in patients with psychotic disorders. Electrophysiologically, Voss et al. (2014) have identified low gamma activity around 25 Hz to promote control in dreams and activity around 40 Hz to induce insight. As to dissociation in dreams, such information is currently not available.

In conclusion, it appears that while lucid dreaming bears high similarity to insightful psychosis, lucid dreaming *per se* seems to be more complex. Our experimental data indicate that frontal lobe activation in consciousness-relevant fast frequencies may be responsible for the perceived changes in dream perception, such as awareness of awareness and dream plot control. Regarding dissociation, we look forward to future research on its physiological correlates and its modulating function in lucid dreams as well as in psychosis.

## Author Contributions

All authors were involved in the preparation of this manuscript.

## Conflict of Interest Statement

The authors declare that the research was conducted in the absence of any commercial or financial relationships that could be construed as a potential conflict of interest.

## Footnotes

1 Note here that our definition of dissociation does not include absorption which would rather be viewed as a component of “sense of reality” (see Voss et al., 2013).

2 Interestingly, Friston (2010) and Carhart-Harris et al. (2014) have associated primary states of consciousness with high entropy and attenuated criticality. Hightened entropy is associated with increased subjective uncertainty and surprise or “puzzlement” (p. 2). According to Friston’s (2010) free-energy principle, the brain aims to keep surprise and free energy at a minimum. Interestingly, although not widely investigated, either surprise or uncertainty are rarely mentioned in dream reports (Merritt et al., 1994; Hobson and Friston, 2012) but both might be more frequent at the onset of lucidity and in prelucid dreams – which are characterized by uncertainty as to whether one is now dreaming. By contrast, both are often reported by psychotic patients, suggesting that dreams may share similarities with psychoses but that the cost in terms of entropy and free energy elevations are less profound in dreams compared to psychosis, probably owing to neurophysiologic state-characteristics.

3 The patient was extensively informed about the procedure and gave written consent to the treatment attempt. According to guidelines pertaining to individual treatment attempts, an ethics approval was not obtained.https://www.med.uni-goettingen.de/de/media/V2_ethikkommitee/Informationsblatt_Individuelle_Heilversuche.pdf. Specifically, an individual treatment attempt does not require an ethics approval provided that the patient is informed about the procedure, gives written consent and the procedure itself (tACS) is not completely new. All of these recommendations were applicable in this case.
